# Prevalence of Psychoactive Substance Use and Violent Death: Toxicological and Geospatial Evidence from a Four-Metropolitan-Area Cross-Sectional Study in Brazil

**DOI:** 10.3390/toxics14010103

**Published:** 2026-01-22

**Authors:** Henrique Silva Bombana, Vanderlei Carneiro da Silva, Ivan Dieb Miziara, Heráclito Barbosa Carvalho, Mauricio Yonamine, Vilma Leyton

**Affiliations:** 1 Departamento de Análises Clínicas e Toxicológicas, Faculdade de Ciências Farmacêuticas FCF, Universidade de São Paulo, São Paulo 05508-000, SP, Brazil; yonamine@usp.br; 2 Clinical and Epidemiological Research Center, Universitary Hospital, University of São Paulo, São Paulo 05508-000, SP, Brazil; vnd.crn@gmail.com; 3 Departamento de Medicina Legal, Bioética, Medicina do Trabalho e Medicina Física e Reabilitação Faculdade de Medicina FMUSP, Universidade de São Paulo, São Paulo 01249-903, SP, Brazil; ivan.miziara@usp.br (I.D.M.); vileyton@usp.br (V.L.); 4 Departamento de Medicina Preventiva, Faculdade de Medicina FMUSP, Universidade de São Paulo, São Paulo 01249-903, SP, Brazil; heracc@usp.br

**Keywords:** post-mortem, violent deaths, illicit drugs, homicides

## Abstract

External causes account for over four million deaths globally each year, with psychoactive substance use being a major risk factor. However, the true impact and regional patterns of psychoactive substance use in these deaths remains undefined in Brazil. To address this critical knowledge gap, this pioneering four-city study sought to elucidate the prevalence of alcohol and drug use by external cause victims. We collected postmortem blood from 3577 victims of violent death across four distinct Brazilian cities (Belém, Recife, Vitória, and Curitiba), representing the North, Northeast, Southeast, and South regions, respectively, using a standardized protocol to identify alcohol, illicit drugs, and psychoactive medicines. Analysis revealed a predominantly male cohort (89.7%; 56.0% aged 30 years or more), with homicide as the primary manner of death (67.3%). Over half of the victims (53.0%) tested positive for at least one psychoactive substance prior to death; cocaine (29.6%) and alcohol (27.7%) were most common. Substance use was highest among homicide victims (55.7%), especially cocaine (36.0%), and among self-harm cases (54.6%), which showed elevated benzodiazepine prevalence (20.0%). Substance use patterns varied regionally: alcohol-related deaths were more common in Recife (Northeast), drug-only deaths concentrated in Vitória (Southeast) and Belém (North), and Curitiba (South) showed a higher prevalence of alcohol use versus drug use. This widespread, regionally heterogeneous prevalence underscores the urgent need for targeted, region-specific interventions. By critically linking psychoactive substance use to various modes of violent death, these data provide crucial forensic and public health insights to inform tailored preventive strategies.

## 1. Introduction

External causes of mortality refer to unnatural deaths not related to diseases or pathological conditions, as defined in chapter 23 of the 11th revision of the International Classification of Diseases (ICD-11). These include injuries from various external agents and are categorized by the World Health Organization (WHO) into unintentional (accidental), intentional, interpersonal, self-inflicted, legal intervention, war, civil unrest, and undetermined intent [1] and are typically referred to as violent deaths.

In 2021, external causes accounted for over 4.4 million deaths globally, with traffic crashes leading at more than 1.1 million fatalities [1]. In Brazil, these causes resulted in 159,534 deaths in 2024, ranking fourth after circulatory system diseases, neoplasms, and respiratory diseases. Mortality rates from external causes have remained relatively stable from 2012 to 2023, with approximately 75 deaths per 100,000 inhabitants in 2023 [2]. According to the Pan American Health Organization (PAHO), traffic crashes combined with interpersonal violence and self-harm prematurely cost Brazil more than 3 million years of life and accounted for a loss of 3.3 million Disability-Adjusted Life Years (DALYs) [3].

External causes remain a major public health concern in Brazil, with an age-adjusted mortality rate of approximately 70.8 per 100,000 population in 2023, reflecting significant losses due to homicides (31.6/100,000), traffic crashes (15.1/100,000), and suicides (6.4/100,000). Mortality disproportionately affects men and young adults, while regional disparities show higher risks in the North, Northeast, and Central-West regions [2]. These deaths not only cause premature mortality but also contribute substantially to the nation’s economic and social burden.

Several complex factors mediate injuries from external causes, complicating the understanding of their pathways. Alcohol and drug use stand out as significant risk factors for such injuries and deaths. Goldstein (1985) [4] proposed a triadic framework to understand the violence-drug nexus: psychopharmacological effects of substances (e.g., alcohol, cocaine, stimulants) that may provoke violent behavior; economic-compulsive motives driven by drug-seeking; and systemic factors related to the illegal drug market, which primarily victimizes users. These systemic factors stem from the illicit nature of drugs, rendering violence endemic to their context [4].

Victimization from external causes may also be linked to the use of psychoactive substances (PSAs), since it impairs cognitive and motor functions, potentially leading to involvement in accidents or acts that put the physical safety of the user or others at risk. These effects are generally associated with the pharmacological classes of the PSAs and dose-dependent [5].

Brazil’s continental size encompasses diverse social, economic, cultural, and health landscapes, leading to regional variations in PSA use patterns and their impact on deaths from external causes. Regional differences also affect drug availability and pricing, influencing consumer profiles [6]. Despite the significance of substance use in external cause mortality, Brazil lacks nationwide studies correlating PSAs use with deaths from traffic crashes, homicides, and suicides. Amid varying patterns of substance use and drug availability across Brazil, a nationwide study correlating PSA use to these violent deaths remains crucial but methodologically challenging. This study aims to fill this knowledge gap by pioneering a comprehensive assessment across four metropolitan-regions.

## 2. Materials and Methods

### 2.1. Study Design and Sample Collection

Deceased victims from external causes were included in a cross-sectional study in four Brazilian metropolitan areas. In each geographical region, one state capital was selected as a target based on mortality rate and for being located along drug trafficking routes. Belém/PA, Recife/PE, Vitória/ES, and Curitiba/PR were involved in sample collection representing the North, Northeast, Southeast and South regions, respectively. In addition to the capital cities, satellite cities from each metropolitan region were also included. The selected cities exhibit significant differences in cultural, economic, social, and sanitary aspects, with human development indices (HDIs) comparable to those of different countries, such as Tunisia (0.746), South Africa (0.741), Qatar (0.886), and Portugal (0.890) (Table 1). Sampling occurred during autopsies at the Institutes of Legal Medicine (ILM), which are official public forensic institutions responsible for autopsies in criminal cases.

A representative sample of all violent death cases in each city was achieved by adopting a probabilistic sampling strategy [7]. All days and times were proportionally represented through the use of a structured sampling schedule, in which data collection occurred on alternating days, within the same time period, over the course of two weeks, until all days of the week were included. Subsequently, the sequence of weekdays was repeated during the alternate-day periods. This approach ensured the acquisition of a population-based sample for each metropolitan region included in the study. Sampling occurred in the four cities simultaneously and continuously from March 2022 to June 2024. All victims older than 18 years who were autopsied within eight hours of death were included. Decomposed corpses and victims who received six or more hours of medical treatment were excluded.

Data from the victim (age and sex) and information about the fatal event (i.e., day, time, cause and manner of death) were obtained from police records. Only closed cases with definitively determined causes of death were included.

### 2.2. Toxicological Analysis

Cardiac blood samples (8 mL) were collected during autopsies, using vacuum tubes containing sodium fluoride and ethylenediamine tetraacetic acid (EDTA) and stored at −20 °C until transportation to the Toxicology Laboratory at the University of Sao Paulo Medical School. In cases where the cardiac chamber was not intact and sample risked contamination with stomach contents or other biological fluids or if there was insufficient blood due to the extent of the injuries, peripheral (femoral) blood was collected.

All blood samples were analyzed at the Toxicological Laboratory from the University of Sao Paulo Medical School. Blood Alcohol Concentration (BAC) was analyzed by headspace gas chromatography with a flame ionization detector (HS-GC-FID) from Thermo Fisher Scientific (Waltham, MA, USA) whilst drugs were detected by liquid chromatography with tandem mass spectrometry (LC-MS/MS) from Shimadzu Corporation (Kyoto, Japan). All analyzed substances and their respective cutoffs are presented in Table 2. Analytes were chosen given its importance at a Brazilian setting. Benzoylecgonine, a major cocaine metabolite, was used to assess cocaine consumption.

Substance use was categorized into five groups: (1) Any alcohol presence—detection of alcohol; (2) at least one substance—confirmed positivity for alcohol and/or other drugs; (3) only alcohol—positive BAC but negative for all other drugs; (4) only other drugs—negative BAC but positive for at least one other drug; (5) two or more substances—positive for at least two different substances (which may include alcohol).

Additionally, proportions for alcohol-positive cases and drug-positive cases are reported, along with other combinations. These proportions indicate positivity for each respective group of substances and do not mutually exclude the concurrent use of other substances.

### 2.3. Geospatial Analysis

A geospatial analysis based on the macro region within the state capital was conducted to study the influence of alcohol and other drug use according to the place of the fatal injury. Death densities were calculated for each of the 4 macro regions based on the population rate for 2021 (per 100,000 population).

A map of Brazil divided into all states was used to input data on death densities by states according to drug and alcohol use positivity. A color gradient was used to illustrate death density rates across all four included states, with darker tones representing higher densities than lighter ones. The grouping of density rates across maps was reached using a Jerks algorithm, which makes an approximation of the intervals to the closest possible value of the median for each rate reported. MapInfo Professional software, version 10.0 was used.

Additionally, a ratio was created between mean death densities obtained for drug and alcohol positivity (ρ drug positivity/ρ alcohol positivity) grouped according to the four Brazilian cities involved in the study. A city with a “drug problem ratio” (DPR) greater than 1 indicates a higher density of drug positivity compared to alcohol, whereas rate values less than 1 indicate the opposite. DPR I is based on any results of alcohol and drugs, whereas DPR II considers findings of alcohol alone and drugs alone.

### 2.4. Statistical Analysis

Absolute and relative frequencies (%) are presented for categorical variables. Associations between categorical variables were analyzed using bivariate cross-tabulation and Pearson’s chi-square test. Crude odds ratios (ORs) and adjusted odds ratios (aORs), adjusting for sex, use of alcohol, cocaine, cannabis, and benzodiazepines, were calculated using multivariable logistic regression. Variables for each logistic regression model were selected based upon statistical significance derived from Pearson’s chi-square test. Significance levels were set at *p* < 0.05. All analyses were conducted using STATA statistical software, version 16.1 (College Station, TX, USA).

## 3. Results

**Figure 1:**
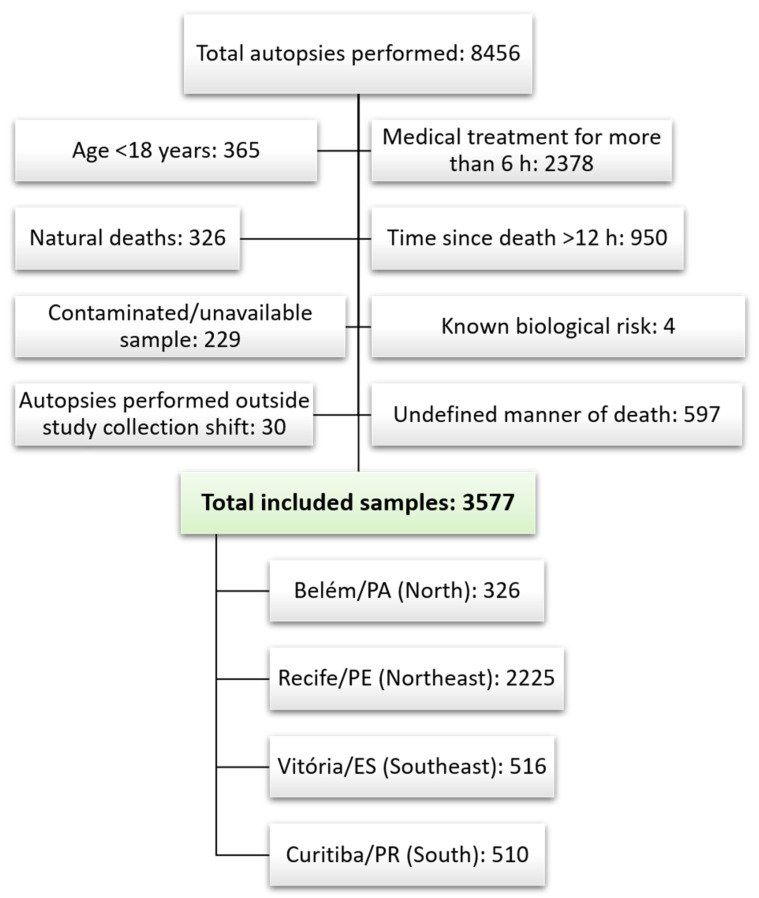
Flowchart of sample inclusion and exclusion criteria and total number of samples by included cities.

A total of 3577 victims were included (Figure 1). The vast majority were male (89.7%), aged 30 years or older (56.0%), and non-white (80.8%). Homicides were the main manner of death (67.3%), followed by traffic crashes (14.7%) and suicides (9.2%). More than half of the victims (53.0%) had consumed at least one substance before death, with cocaine being the most common (29.6%), followed by alcohol (27.7%), benzodiazepines (6.8%), and cannabis (2.2%). Homicide victims had the highest prevalence of alcohol/drug use (55.7%; 95% CI: 53.7–57.7), mainly cocaine use (36.0%; 95% CI: 34.1–38.0). Self-harm victims had the second-highest prevalence of substance use, with alcohol being the main substance (29.4%; 95% CI: 24.7–34.6). Those victims also showed very high rates of benzodiazepine consumption; one-fifth of the sample tested positive in toxicological analysis. While more than two in five traffic-related victims had consumed substances, this was driven especially by alcohol (38%; 95% CI: 33.9–42.2) (Table 3).

Men were more likely to have consumed substances (OR = 1.52; 95% CI: 1.24–1.89), especially drugs (OR = 1.92; 95% CI: 1.42–2.61) or a combination of substances. Young victims (less than 30 years old) consumed more drugs (OR = 1.89; 95% CI: 1.59–2.22) while those aged 30 or more were more likely to have consumed alcohol alone (OR = 1.94; 95% CI: 1.59–2.36). Alcohol alone was strongly associated with traffic-related deaths, drug use was associated with homicides, and a combination of substances was associated with homicides and suicides. Alcohol use was associated with deaths occurring at nighttime and at weekends, used alone or in combination with other substances (Table 4).

**Figure 2:**
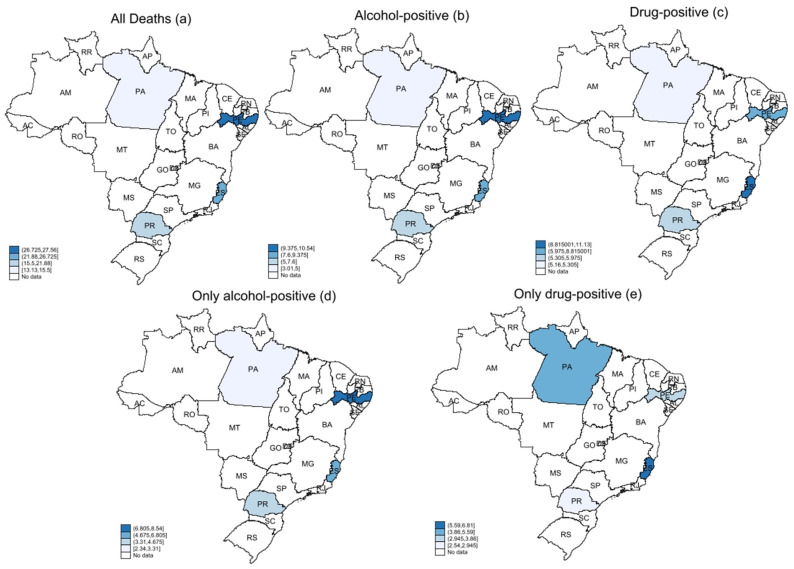
Choropleth maps of Drug Problem Ratios (DPRs) for all deaths, alcohol-positive, drug-positive, alcohol-only, and drug-only cases across four Brazilian metropolitan areas. For visual clarity, the entire state is shaded even though data were collected only in the respective state capital.

The use of substances across the four cities was uneven. Deaths involving alcohol, either solely or in combination with drugs, were more prevalent in Recife/PE (Northeast), while deaths involving only drugs were concentrated in Vitória/ES (Southeast) and Belém/PA (North) (Figure 2). The calculated DPR further confirmed the prominence of drug-related deaths in these two capitals. In contrast, Curitiba/PR (South) showed a higher prevalence of alcohol-related deaths compared to drug-related deaths, whereas Recife showed a more similar distribution between alcohol- and drug-related deaths (Table 5).

## 4. Discussion

The present study represents the first investigation among four Brazilian regions, represented by four state capitals and its metropolitan areas, to comprehensively examine the association between psychoactive substance use and violent deaths. The finding that over half of violent death victims (53.0%) tested positive for at least one psychoactive substance prior to death underscores the substantial role of substance use in premature mortality from external causes in Brazil. Although causal relationships cannot be established in cross-sectional studies, our findings highlight a significant issue regarding the association between psychoactive substance use and deaths from external causes. Overall, our findings align with international [8,9,10] and national [7,11,12] findings regarding postmortem toxicological analysis, while also revealing important regional and substance-specific patterns. Internationally, patterns of substance use vary by region. For example, in Cape Town, South Africa, 61% of assessed victims had used drugs, with methamphetamine, methaqualone, and diphenhydramine being the most prevalent [8]. In New South Wales, Australia, among homicide victims, 32.8% tested positive for PSAs, with cannabis (21.4%) and opioids (11.2%) being the most common [9]. By contrast, in Milan, Italy, cocaine was the most frequently used substance (27.9%) among postmortem cases, a pattern more closely aligned with our findings [10].

A previous Brazilian study found considerably higher figures for cocaine and cannabis use among homicide victims, 72.2% and 67.7%, respectively. However, in that study, toxicological analyses were performed in a non-systematic way, restricted to victims in whom there was already some suspicion of drug use [12], which likely led to an overestimation of drug positivity when compared with the population-based and standardized approach used here.

The demographic characteristics of the victims in our study support the evidence that men are more likely not only to use alcohol and other drugs, but also to be involved in injurious events that result in fatal outcomes [13]. Moreover, given the significant association between exclusive drug use and victims under 30 years of age, there is a clear need to identify and intervene on problematic drug use in youth, since such behaviors can lead both to the perpetration of violence and to victimization [14].

Although cannabis is the most widely used drug in Brazil and its consumption has increased in the last decade [15], the prevalence of THC detection in the present study was lower than expected. This is particularly evident when compared with another investigation using a similar study design, which found a higher percentage of THC identification (14.0%) among victims of violent deaths in the city of São Paulo [7]. A direct relationship between cannabis use and violence is not firmly established in the scientific literature. There is ongoing debate about the magnitude of the association between the psychopharmacological effects of cannabis and the occurrence of injuries and deaths. Available data indicate that there may be a moderate increase in the probability of violence among cannabis users, a relationship that seems more evident among those with daily use and some degree of psychosis [16]. The pharmacological effects of cannabis include an altered state of consciousness and changes in time perception; at moderate concentrations, some studies suggest a temporary inhibition of violent behaviors [17]. The decision not to analyze THCCOOH (a THC metabolite) was based on our aim to capture acute use of psychoactive substances, as the presence of THCCOOH may reflect past rather than recent cannabis use. Moreover, available evidence indicates only low to moderate postmortem redistribution of THC [18], suggesting that PMR is unlikely to explain the low THC detection rate observed in our samples. Our results therefore do not suggest an absence of cannabis use in the population, but rather a low frequency of THC detection among victims of external causes in the studied cities.

Cocaine, in contrast, emerged as the most frequently detected substance in our sample, particularly among homicide victims. This pattern is consistent with Brazil’s role as both a transit corridor and the largest consumer market for cocaine in South America. Previous studies have identified strong associations between cocaine use, criminal activity, and violent victimization [19]. Given that this is a cross-sectional study, it cannot establish causality between cocaine use and homicide. The observed associations should be interpreted in light of psychopharmacological effects, economic-compulsive motives, and systemic violence related to the illegal drug market rather than attributing them to acute intoxication alone. Furthermore, cocaine consumption has been associated with increased criminal activities such as robberies [20].

Despite regulation since 1998, alcohol remains the main contributor to traffic crashes in Brazil. It reduces visual acuity, alertness, and motor and cognitive coordination, which are essential for driving [21]. Even at low concentrations, alcohol causes impairments in users, and there is no safe level for driving, since there is a dose–response relationship with an exponential increase in the risk of involvement in traffic crashes [22]. In legal terms, Brazil has one of the strictest frameworks in the world, adopting a zero-tolerance policy for alcohol in drivers. In spite of zero-tolerance framework in Brazilian law, there are two approaches to drunk drivers: an administrative one (BAC < 0.6 g/L), in which the driver receives a fine and loses the driving license, and a criminal one (BAC ≥ 0.6 g/L), in which the driver is arrested in addition to the administrative sanctions. Despite this, alcohol continues to be detected as the main substance among individuals injured [23] and killed [7,24,25] in traffic crashes. Our multicenter postmortem data confirm that alcohol remains the primary substance associated with traffic-related deaths. Enforcement of alcohol and other drug use among drivers is one of the most effective ways to reduce consumption of these substances by drivers and, consequently, to reduce deaths resulting from alcohol use. On the other hand, under current Brazilian law, drivers may refuse to undergo breathalyzer testing, which, even with administrative sanctions (loss of driver’s license and fines), results in a certain degree of impunity for offenders [26]. Thus, to translate Brazil’s strict legal framework into greater population impact, it will be necessary to strengthen random testing and police enforcement, reduce the incentive to refuse testing, and integrate roadside enforcement with broader alcohol-control policies.

Regarding the geospatial analysis of alcohol and drug use, Belém /PA (North) and Vitória/ES (Southeast) showed the highest Drug Problem Ratio coefficients, indicating greater involvement of drugs compared with alcohol in violent deaths in these capitals. In our study, the drug problem ratio reflected the relationship between death densities with drug positivity and those with alcohol positivity, thereby summarizing whether a region is more “drug-dominant” or “alcohol-dominant” in terms of substance involvement in violent deaths. That is, a value greater than 1.0 indicates that deaths in the city are more strongly related to the use of drugs other than alcohol, whereas values below 1.0 indicate a stronger relationship between deaths and alcohol consumption. Belém lies within the Amazon region and has several river branches used to transport cocaine from neighboring countries such as Peru and Colombia, which may partly explain the higher drug consumption relative to alcohol in this area [27]. By contrast, Recife/PE (Northeast) and Curitiba/PR (North) are also located along drug trafficking routes but showed higher alcohol use than drug use [6]. These patterns suggest that, beyond trafficking routes, local drug markets, cultural norms, and social context shape which substances are most involved in fatal events. Therefore, more in-depth studies are needed to understand the dynamics of alcohol and other drug use in these cities and regions, ideally combining toxicological data with information on seizures, prices, treatment demand, and community-level use.

Despite declines in some states, Brazil continues to face high homicide rates. In 2024, over 39,000 deaths resulted from homicides nationwide. Geographic distribution is highly uneven: Northeastern states report the highest rates, whereas the Southeast and South have seen notable declines. Furthermore, regions characterized by intense drug trafficking, especially along international borders, tend to exhibit elevated rates. Beyond drug-trade violence, cocaine consumption itself may contribute to the heightened mortality in specific regions. Our findings substantiate a link between cocaine use and homicides, implying that increased availability and use of cocaine correlate with heightened lethal violence. This connection is reinforced by a previous Brazilian study, which found that victims of violent deaths with a prior criminal record were more likely to have consumed psychoactive substances [7], supporting the hypothesis of a mutually reinforcing cycle between substance use, criminal involvement, and victimization in vulnerable populations.

A major strength of the study was the use of blood samples for toxicological analysis, since this is one of the best biological matrices to estimate the acute use of substances and that the victim could be under the influence at the time of death. In addition, the systematic and standardized collection of samples provided valuable inter-regional insights from different Brazilian locations, allowing a better understanding of the burden of drugs on violent deaths through a population-based analysis over a 27-month period and across four cities. This multicenter, probabilistic sampling approach enhances the external validity of our estimates and allows for meaningful comparisons between manners of death and regions. The inclusion of different Brazilian cities with diverse cultural, social, economic, and health contexts represents an important strength of this study. The findings may be generalizable to other Brazilian cities with similar HDI levels and even to countries that share comparable social and economic characteristics. 

Despite these strengths, the study has several limitations. Because the locations were selected based on mortality rate data, this could introduce selection bias. Nevertheless, the inclusion of cities with diverse social profile enhances the potential for generalization to other Brazilian settings. Moreover, even though clinically relevant, the lack of medical information in suicide cases makes it impossible to assess whether the benzodiazepines identified were being used under medical supervision or deliberately without professional monitoring. Furthermore, the strategy of excluding cases with more than six hours of medical treatment may have contributed, for example, to the low number of cases resulting from traffic crashes. Thus, the low count here should not be interpreted as reflecting low traffic mortality rates in these cities. Additionally, post-mortem redistribution (PMR), the diffusion-driven movement of substances between organs and body cavities after death, may have influenced the results, as most samples consisted of cardiac blood. We recognize that this choice could have introduced some bias. However, cardiac blood was selected because it allowed standardized sample collection across all participating cities. Moreover, the inclusion of only relatively “fresh” corpses likely minimized the extent of PMR in our samples.

As noted above, the cross-sectional design of the study also prevents establishing a causal relationship between drug use and deaths, and toxicological positivity cannot be equated with a direct cause of the fatal event. Nevertheless, evidence points to a higher probability of victimization after the consumption of psychoactive substances, a fact supported by the present findings. Therefore, drug use is a reality in the Brazilian population that must be approached holistically so that the associated morbidity and mortality can be effectively controlled through a combination of prevention, harm reduction, treatment expansion, and criminal-justice reform.

The relationship between deaths from external causes and drug use was evident in the data presented in this study, with more than half of the victims having used psychoactive substances, mainly cocaine and alcohol. Despite extensive debate, alcohol remains the main actor in traffic-related deaths, whereas cocaine is more associated with homicides, and benzodiazepines are more associated with suicides. The prohibitionist, criminalizing model of drug control creates parallel and often violent markets, which can contribute to drug-related mortality [28] and to environments characterized by systemic and structural violence, in addition to the racial and social injustices intrinsically associated with this perspective [29]. There is, therefore, an urgent need to address drug consumption from a public health standpoint [30], recognizing that policies which prioritize treatment and social protection over punitive responses are more likely to reduce both the substance-related harm and violent deaths. In addition, the geospatial patterns observed in this study, with Belém (PA) and Vitória (ES) showing higher relative involvement of illicit drugs, and Recife (PE) and Curitiba (PR) showing a more prominent role of alcohol, illustrate the heterogenous burden of psychoactive substances on violent deaths across the country. These regional differences suggest that prevention and control strategies must be tailored to local profiles of use and market dynamics, rather than relying on a single national model.

## 5. Conclusions

The use of psychoactive substances is frequent among victims of violent deaths in Brazil, with more than half of the victims having consumed them, mainly cocaine and alcohol. Regional patterns also differed: higher alcohol-related death densities in Recife (Northeast), higher drug-related densities in Vitória (Southeast) and Belém (North), and relatively greater alcohol involvement in Curitiba (South). Together, toxicological and geospatial analyses provide a crucial evidence base to guide region-specific, public-health-oriented responses.

## Figures and Tables

**Table 1:** Cities included in the study, their Human Development Index (HDI), and mortality rates from external causes.

City	State	Brazilian Geographical Region	HDI *	Mortality Rate from External Causes/100,000 *
Belém	Pará (PA)	North	0.746	43.5
Recife	Pernambuco (PE)	Northeast	0.772	143.8
Vitória	Espirito Santo (ES)	Southeast	0.845	197.0
Curitiba	Paraná (PR)	South	0.885	80.4

* data from 2021.

**Table 2:** Psychoactive substances included in the toxicological analysis and corresponding analytical cutoff concentrations in postmortem blood.

Analyte	Cutoff (ng/mL)
Alcohol (g/L)	0.2
Cannabis	
∆9-tetrahydrocannabinol (THC)	1
Cocaine and metabolites	
Cocaine	20
Benzoylecgonine	40
Cocaethylene	20
Anhydroecgonine methyl ester (AEME—crack cocaine)	20
Amphetamines	
Amphetamine	20
Methamphetamine	20
MDMA (3,4-methylenedioxymethamphetamine—ecstasy) MDA (3,4-methylenedioxyamphetamine)	20
	20
Benzodiazepines	
Clonazepam	5
7-aminoclonazepam	5
Alprazolam	5
Diazepam	10
Bromazepam	20
Oxazepam	10

**Table 3:** Prevalence of substance use among victims of violent deaths, overall and by manner of death (traffic-related, homicide, suicide, poisoning and other).

	All Deaths		Traffic-Related		Homicides		Suicides		Poisoning		All Others	
	%	(95% CI)	%	(95% CI)	%	(95% CI)	%	(95% CI)	%	(95% CI)	%	(95% CI)
*n*	3577		524		2406		330		52		265	
**General positivity**												
At least one	53.0	(51.3–54.6)	46.0	(41.8–50.2)	55.7	(53.7–57.7)	54.6	(49.1–60.0)	65.4	(51.3–77.2)	37.7	(32.1–43.7)
Only alcohol	16.6	(15.4–17.9)	30.9	(27.1–35.0)	14.5	(13.2–16.0)	14.5	(11.1–18.8)	5.8	(1.8–16.8)	12.5	(8.9–17.0)
Only drugs	21.6	(20.3–23.0)	5.5	(3.9–7.9)	27.3	(25.5–29.1)	10.9	(7.9–14.8)	32.7	(21.2–46.8)	12.8	(9.3–17.5)
Two or more	10.9	(9.9–12.0)	7.1	(5.2–9.6)	11.7	(10.5–13.1)	14.8	(11.4–19.1)	13.5	(6.4–26.0)	6.0	(3.7–9.6)
**Specific positivity**												
Alcohol	27.7	(26.2–29.2)	38.0	(33.9–42.2)	26.4	(24.7–28.2)	29.4	(24.7–34.6)	19.2	(10.5–32.5)	18.5	(14.2–23.7)
Cocaine	29.6	(28.1–31.1)	9.9	(7.6–12.8)	36.0	(34.1–38.0)	21.2	(17.1–26.0)	44.2	(31.2–58.1)	17.7	(13.6–22.8)
Cannabis	2.2	(1.2–2.7)	0	-	3.2	(2.5–4.0)	0	-	0	-	-	-
Benzodiazepines	6.8	(6.0–7.7)	4.4	(2.9–6.5)	4.8	(4.0–5.8)	20.3	(16.3–25.0)	25.0	(14.9–38.8)	9.0	(6.1–13.2)
Alcohol + Cocaine	9.8	(8.8–10.7)	5.2	(3.6–7.4)	10.9	(9.7–12.2)	11.5	(8.5–15.4)	13.5	(6.4–26.0)	5.3	(3.1–8.7)
Alcohol + Cannabis	0	-	0	-	0	-	0	-	0	-	0	
Alcohol + Benzodiazepines	1.7	(1.4–2.2)	1.9	(1.0–3.5)	1.3	(1.0–1.9)	4.2	(2.5–7.0)	3.9	(1.0–14.5)	1.5	(1.0–4.0)

Note: - = <0.01.

**Table 4:** Associations between substance-use patterns and demographic/fatal-event characteristics among victims of violent deaths.

	All Victims		At Least One		Any Alcohol		Only Alcohol		Only Drugs		Two or More	
	%	(95% CI)	OR	(95% CI)	OR	(95% CI)	OR	(95% CI)	OR	(95% CI)	OR	(95% CI)
**Sex**												
Women	10.9	(9.9–11.9)	1.0		1.0		1.0		1.0		1.0	
Men	89.1	(88.1–90.1)	1.52	(1.24–1.89) *	1.06	(0.85–1.31)	1.16	(0.87–1.57)	1.92	(1.42–2.61) *	1.52	(1.04–2.25) *
**Age**												
Less than 30	44.0	(42.3–45.7)	1.0		1.0		1.0		1.0		1.0	
More than 30 or equal	56.0	(54.3–57.8)	1.09	(0.95–1.26)	1.42	(1.23–1.63) *	1.94	(1.59–2.36) *	0.53	(0.45–0.63) *	1.11	(0.89–1.39)
**Race**												
Non-white	80.8	(79.4–82.0)	1.0		1.0		1.0		1.0		1.0	
White	19.2	(18.0–20.6)	1.17	(0.98–1.40)	2.52	(2.12–3.03) *	0.92	(0.72–1.16)	0.82	(0.65–1.03)	1.64	(1.27–2.12) *
**Injury type**												
All others	7.4	(6.6–8.3)	1.0		1.0		1.0		1.0		1.0	
Homicides	67.3	(65.7–68.8)	2.00	(1.51–2.66) *	1.42	(1.06–1.91) *	1.38	(0.93–2.08)	1.98	(1.32–2.95) *	2.18	(1.24–3.83) *
Traffic-related	14.7	(13.5–15.9)	1.31	(0.96–1.80)	2.30	(1.66–3.19) *	3.27	(2.13–5.02) *	0.32	(0.19–0.56) *	1.22	(0.64–2.32)
Suicide	9.2	(8.3–10.2)	1.81	(1.28–2.56) *	2.12	(1.49–3.02) *	1.08	(0.65–1.78)	0.76	(0.45–1.28)	2.70	(1.43–5.06) *
Poisoning	1.4	(1.1–1.9)	2.78	(1.40–5.51) *	1.04	(0.52–2.09)	1.14	(1.07–1.22)	3.71	(1.77–7.75) *	2.62	(0.94–7.27)
**Death time**												
Day	46.1	(44.3–47.9)	1.0		1.0		1.0		1.0		1.0	
Night	53.9	(52.1–55.7)	1.75	(1.50–2.04) *	1.36	(1.17–1.59)	2.00	(1.61–2.48) *	1.12	(0.93–1.35)	1.64	(1.28–2.10) *
**Death day**												
Weekday	62.6	(61.0–64.2)	1.0		1.0		1.0		1.0		1.0	
Weekend	37.4	(35.8–39.0)	1.52	(1.32–1.76)	1.52	(1.31–1.76) *	2.28	(1.89–2.76) *	0.84	(0.70–1.01)	1.41	(1.13–1.78) *

* *p* < 0.005.

**Table 5:** Mean death rates by substance-positivity group and Drug Problem Ratio (DPR) across Brazilian regions included in the study.

City	All Deaths		Alcohol-Positive		Drug-Positive		Only Alcohol		Only Drugs		DPR I	DPR II
	Mean	(95% CI)	Mean	(95% CI)	Mean	(95% CI)	Mean	(95% CI)	Mean	(95% CI)		
Belém/PA—North	13.13	(5.48–20.78)	3.01	(1.55–4.48)	5.16	(1.12–9.20)	2.34	(1.03–3.66)	4.37	(0.66–8.09)	1.57	1.95
Recife/PE—Northeast	27.56	(21.90–33.23)	10.54	(7.92–13.15)	6.50	(4.62–8.38)	8.54	(6.41–10.67)	3.35	(2.26–4.44)	0.80	0.57
Vitória/ES—Southeast	25.89	(21.77–30.01)	8.21	(6.21–10.21)	11.13	(8.05–14.20)	5.07	(2.51–7.63)	6.81	(3.47–10.16)	1.39	1.39
Curitiba/PR—South	17.87	(13.85–21.88)	6.99	(4.69–9.29)	5.45	(3.50–7.41)	4.28	(2.07–6.49)	2.54	(1.60–3.49)	1.05	1.09

## Data Availability

Since this study used data from police reports, even anonymized, to ensure the integrity of the police data, and given that cases may still be under judicial review, the database will not be publicly available. Any questions related to the presented data can be addressed to the corresponding author (HSB: hbombana@usp.br), who will evaluate the possibility of providing any data that does not interfere with ongoing police investigations.

## Abbreviations

The following abbreviations are used in this manuscript:

BAC	Blood alcohol concentration
DPR	Drug problem ratio
HDI	Human development index
OR	Odds ratio
PSA	Psychoactive substances
THC	Δ9-tetrahydrocannabinol
